# Supplementary material for: A fault-intrusion-tolerant system and deadline-aware algorithm for scheduling scientific workflow in the cloud
Source: PeerJ Comput Sci. 2021 Nov 2;7:e747. doi: 10.7717/peerj-cs.747 (PMC8576554; doi:10.7717/peerj-cs.747)
Supplement: Supplemental Information 6 [file peerj-cs-07-747-s006.zip › Myproject1.1/docs/org/cloudbus/cloudsim/DatacenterCharacteristics.html]

DatacenterCharacteristics


JavaScript is disabled on your browser.


- Overview
- Package
- Class
- Use
- Tree
- Deprecated
- Index
- Help

- Prev Class
- Next Class

- Frames
- No Frames

- All Classes

- Summary:
- Nested |
- Field |
- Constr |
- Method

- Detail:
- Field |
- Constr |
- Method


org.cloudbus.cloudsim

## Class DatacenterCharacteristics

- java.lang.Object
- - org.cloudbus.cloudsim.DatacenterCharacteristics

- ---

    

  ```
  public class DatacenterCharacteristics
  extends java.lang.Object
  ```

  DatacenterCharacteristics represents static properties of a resource such as resource
  architecture, Operating System (OS), management policy (time- or space-shared), cost and time
  zone at which the resource is located along resource configuration.

  Since:
  :   CloudSim Toolkit 1.0

- - ### Field Summary

    Fields

    | Modifier and Type | Field and Description |
    | --- | --- |
    | `static int` | `ADVANCE_RESERVATION` A resource that supports Advanced Reservation mechanisms. |
    | `static int` | `OTHER_POLICY_DIFFERENT_RATING` Assuming all PEs in a Machine have the same rating. |
    | `static int` | `OTHER_POLICY_SAME_RATING` Assuming all PEs in all Machines have the same rating. |
    | `static int` | `SPACE_SHARED` Spaced-shared system using First Come First Serve (FCFS) algorithm. |
    | `static int` | `TIME_SHARED` Time-shared system using Round-Robin algorithm. |
  - ### Constructor Summary

    Constructors

    Constructor and Description

    `DatacenterCharacteristics(java.lang.String architecture,
    java.lang.String os,
    java.lang.String vmm,
    java.util.List<? extends Host> hostList,
    double timeZone,
    double costPerSec,
    double costPerMem,
    double costPerStorage,
    double costPerBw)`

    Allocates a new DatacenterCharacteristics object.
  - ### Method Summary

    Methods

    | Modifier and Type | Method and Description |
    | --- | --- |
    | `protected int` | `getAllocationPolicy()` Gets the allocation policy. |
    | `protected java.lang.String` | `getArchitecture()` Gets the architecture. |
    | `double` | `getCostPerBw()` Get the cost to use bandwidth in this resource. |
    | `double` | `getCostPerMem()` Get the cost to use memory in this resource. |
    | `double` | `getCostPerMi()` Gets the cost per Millions Instruction (MI) associated with a resource. |
    | `double` | `getCostPerSecond()` Gets the cost per second. |
    | `double` | `getCostPerStorage()` Get the cost to use storage in this resource. |
    | `double` | `getCpuTime(double cloudletLength, double load)` Gets the CPU time given the specified parameters (only for TIME\_SHARED). |
    | `<T extends Host>  java.util.List<T>` | `getHostList()` Gets the host list. |
    | `Host` | `getHostWithFreePe()` Gets a Machine with at least one empty Pe. |
    | `Host` | `getHostWithFreePe(int peNumber)` Gets a Machine with at least a given number of free Pe. |
    | `int` | `getId()` Gets the id. |
    | `int` | `getMips()` Gets the total MIPS rating, which is the sum of MIPS rating of all machines in a resource. |
    | `int` | `getMipsOfOnePe()` Gets Millions Instructions Per Second (MIPS) Rating of a Processing Element (Pe). |
    | `int` | `getMipsOfOnePe(int id, int peId)` Gets Millions Instructions Per Second (MIPS) Rating of a Processing Element (Pe). |
    | `int` | `getNumberOfBusyPes()` Gets the total number of BUSY PEs for all Machines. |
    | `int` | `getNumberOfFailedHosts()` Gets the current number of failed machines. |
    | `int` | `getNumberOfFreePes()` Gets the total number of FREE or non-busy PEs for all Machines. |
    | `int` | `getNumberOfHosts()` Gets the total number of machines. |
    | `int` | `getNumberOfPes()` Gets the total number of PEs for all Machines. |
    | `protected java.lang.String` | `getOs()` Gets the os. |
    | `java.lang.String` | `getResourceName()` Gets the name of a resource. |
    | `protected double` | `getTimeZone()` Gets the time zone. |
    | `java.lang.String` | `getVmm()` Gets the VMM in use in the datacenter. |
    | `boolean` | `isWorking()` Checks whether all machines of this resource are working properly or not. |
    | `protected void` | `setAllocationPolicy(int allocationPolicy)` Sets the allocation policy. |
    | `protected void` | `setArchitecture(java.lang.String architecture)` Sets the architecture. |
    | `void` | `setCostPerBw(double costPerBw)` Sets cost to use bw cost to use bw. |
    | `void` | `setCostPerMem(double costPerMem)` Sets cost to use memory. |
    | `protected void` | `setCostPerSecond(double costPerSecond)` Sets the cost per second. |
    | `void` | `setCostPerStorage(double costPerStorage)` Sets cost to use storage. |
    | `protected <T extends Host>  void` | `setHostList(java.util.List<T> hostList)` Sets the host list. |
    | `protected void` | `setId(int id)` Sets the id. |
    | `protected void` | `setOs(java.lang.String os)` Sets the os. |
    | `boolean` | `setPeStatus(int status, int hostId, int peId)` Sets the particular Pe status on a Machine. |
    | `protected void` | `setTimeZone(double timeZone)` Sets the time zone. |
    | `protected void` | `setVmm(java.lang.String vmm)` Sets the vmm. |

    - ### Methods inherited from class java.lang.Object

      `clone, equals, finalize, getClass, hashCode, notify, notifyAll, toString, wait, wait, wait`

- - ### Field Detail


    - #### TIME\_SHARED

      ```
      public static final int TIME_SHARED
      ```

      Time-shared system using Round-Robin algorithm.

      See Also:
      :   Constant Field Values


    - #### SPACE\_SHARED

      ```
      public static final int SPACE_SHARED
      ```

      Spaced-shared system using First Come First Serve (FCFS) algorithm.

      See Also:
      :   Constant Field Values


    - #### OTHER\_POLICY\_SAME\_RATING

      ```
      public static final int OTHER_POLICY_SAME_RATING
      ```

      Assuming all PEs in all Machines have the same rating.

      See Also:
      :   Constant Field Values


    - #### OTHER\_POLICY\_DIFFERENT\_RATING

      ```
      public static final int OTHER_POLICY_DIFFERENT_RATING
      ```

      Assuming all PEs in a Machine have the same rating. However, each Machine has different
      rating to each other.

      See Also:
      :   Constant Field Values


    - #### ADVANCE\_RESERVATION

      ```
      public static final int ADVANCE_RESERVATION
      ```

      A resource that supports Advanced Reservation mechanisms.

      See Also:
      :   Constant Field Values
  - ### Constructor Detail


    - #### DatacenterCharacteristics

      ```
      public DatacenterCharacteristics(java.lang.String architecture,
                               java.lang.String os,
                               java.lang.String vmm,
                               java.util.List<? extends Host> hostList,
                               double timeZone,
                               double costPerSec,
                               double costPerMem,
                               double costPerStorage,
                               double costPerBw)
      ```

      Allocates a new DatacenterCharacteristics object. If the time zone is invalid, then by
      default, it will be GMT+0.

      Parameters:
      :   `architecture` - the architecture of a resource
      :   `os` - the operating system used
      :   `vmm` - the virtual machine monitor used
      :   `hostList` - list of machines in a resource
      :   `timeZone` - local time zone of a user that owns this reservation. Time zone should be of
          range [GMT-12 ... GMT+13]
      :   `costPerSec` - the cost per sec to use this resource
      :   `costPerMem` - the cost to use memory in this resource
      :   `costPerStorage` - the cost to use storage in this resource
      :   `costPerBw` - the cost per bw
  - ### Method Detail


    - #### getResourceName

      ```
      public java.lang.String getResourceName()
      ```

      Gets the name of a resource.

      Returns:
      :   the resource name


    - #### getHostWithFreePe

      ```
      public Host getHostWithFreePe()
      ```

      Gets a Machine with at least one empty Pe.

      Returns:
      :   a Machine object or if not found


    - #### getHostWithFreePe

      ```
      public Host getHostWithFreePe(int peNumber)
      ```

      Gets a Machine with at least a given number of free Pe.

      Parameters:
      :   `peNumber` - the pe number

      Returns:
      :   a Machine object or if not found


    - #### getMipsOfOnePe

      ```
      public int getMipsOfOnePe()
      ```

      Gets Millions Instructions Per Second (MIPS) Rating of a Processing Element (Pe). It is
      assumed all PEs' rating is same in a given machine.

      Returns:
      :   the MIPS Rating or if no PEs are exists.


    - #### getMipsOfOnePe

      ```
      public int getMipsOfOnePe(int id,
                       int peId)
      ```

      Gets Millions Instructions Per Second (MIPS) Rating of a Processing Element (Pe). It is
      essential to use this method when a resource is made up of heterogenous PEs/machines.

      Parameters:
      :   `id` - the machine ID
      :   `peId` - the Pe ID

      Returns:
      :   the MIPS Rating or if no PEs are exists.


    - #### getMips

      ```
      public int getMips()
      ```

      Gets the total MIPS rating, which is the sum of MIPS rating of all machines in a resource.

      Total MIPS rating for:
      - TimeShared = 1 Rating of a Pe \* Total number of PEs- Other policy same rating = same as TimeShared- SpaceShared = Sum of all PEs in all Machines- Other policy different rating = same as SpaceShared- Advance Reservation = 0 or unknown. You need to calculate this manually.

      Returns:
      :   the sum of MIPS ratings


    - #### getCpuTime

      ```
      public double getCpuTime(double cloudletLength,
                      double load)
      ```

      Gets the CPU time given the specified parameters (only for TIME\_SHARED). NOTE: The
      CPU time for SPACE\_SHARED and ADVANCE\_RESERVATION are not yet implemented.

      Parameters:
      :   `cloudletLength` - the length of a Cloudlet
      :   `load` - the load of a Cloudlet

      Returns:
      :   the CPU time


    - #### getNumberOfPes

      ```
      public int getNumberOfPes()
      ```

      Gets the total number of PEs for all Machines.

      Returns:
      :   number of PEs


    - #### getNumberOfFreePes

      ```
      public int getNumberOfFreePes()
      ```

      Gets the total number of FREE or non-busy PEs for all Machines.

      Returns:
      :   number of PEs


    - #### getNumberOfBusyPes

      ```
      public int getNumberOfBusyPes()
      ```

      Gets the total number of BUSY PEs for all Machines.

      Returns:
      :   number of PEs


    - #### setPeStatus

      ```
      public boolean setPeStatus(int status,
                        int hostId,
                        int peId)
      ```

      Sets the particular Pe status on a Machine.

      Parameters:
      :   `status` - Pe status, either Pe.FREE or Pe.BUSY
      :   `hostId` - Machine ID
      :   `peId` - Pe id

      Returns:
      :   otherwise (Machine id or Pe id might not be exist)


    - #### getCostPerMi

      ```
      public double getCostPerMi()
      ```

      Gets the cost per Millions Instruction (MI) associated with a resource.

      Returns:
      :   the cost using a resource


    - #### getNumberOfHosts

      ```
      public int getNumberOfHosts()
      ```

      Gets the total number of machines.

      Returns:
      :   total number of machines this resource has.


    - #### getNumberOfFailedHosts

      ```
      public int getNumberOfFailedHosts()
      ```

      Gets the current number of failed machines.

      Returns:
      :   current number of failed machines this resource has.


    - #### isWorking

      ```
      public boolean isWorking()
      ```

      Checks whether all machines of this resource are working properly or not.

      Returns:
      :   if all machines are working, otherwise


    - #### getCostPerMem

      ```
      public double getCostPerMem()
      ```

      Get the cost to use memory in this resource.

      Returns:
      :   the cost to use memory


    - #### setCostPerMem

      ```
      public void setCostPerMem(double costPerMem)
      ```

      Sets cost to use memory.

      Parameters:
      :   `costPerMem` - cost to use memory


    - #### getCostPerStorage

      ```
      public double getCostPerStorage()
      ```

      Get the cost to use storage in this resource.

      Returns:
      :   the cost to use storage


    - #### setCostPerStorage

      ```
      public void setCostPerStorage(double costPerStorage)
      ```

      Sets cost to use storage.

      Parameters:
      :   `costPerStorage` - cost to use storage


    - #### getCostPerBw

      ```
      public double getCostPerBw()
      ```

      Get the cost to use bandwidth in this resource.

      Returns:
      :   the cost to use bw


    - #### setCostPerBw

      ```
      public void setCostPerBw(double costPerBw)
      ```

      Sets cost to use bw cost to use bw.

      Parameters:
      :   `costPerBw` - the cost per bw


    - #### getVmm

      ```
      public java.lang.String getVmm()
      ```

      Gets the VMM in use in the datacenter.

      Returns:
      :   the VMM name


    - #### getId

      ```
      public int getId()
      ```

      Gets the id.

      Returns:
      :   the id


    - #### setId

      ```
      protected void setId(int id)
      ```

      Sets the id.

      Parameters:
      :   `id` - the new id


    - #### getArchitecture

      ```
      protected java.lang.String getArchitecture()
      ```

      Gets the architecture.

      Returns:
      :   the architecture


    - #### setArchitecture

      ```
      protected void setArchitecture(java.lang.String architecture)
      ```

      Sets the architecture.

      Parameters:
      :   `architecture` - the new architecture


    - #### getOs

      ```
      protected java.lang.String getOs()
      ```

      Gets the os.

      Returns:
      :   the os


    - #### setOs

      ```
      protected void setOs(java.lang.String os)
      ```

      Sets the os.

      Parameters:
      :   `os` - the new os


    - #### getHostList

      ```
      public <T extends Host> java.util.List<T> getHostList()
      ```

      Gets the host list.

      Type Parameters:
      :   `T` - the generic type

      Returns:
      :   the host list


    - #### setHostList

      ```
      protected <T extends Host> void setHostList(java.util.List<T> hostList)
      ```

      Sets the host list.

      Type Parameters:
      :   `T` - the generic type

      Parameters:
      :   `hostList` - the new host list


    - #### getTimeZone

      ```
      protected double getTimeZone()
      ```

      Gets the time zone.

      Returns:
      :   the time zone


    - #### setTimeZone

      ```
      protected void setTimeZone(double timeZone)
      ```

      Sets the time zone.

      Parameters:
      :   `timeZone` - the new time zone


    - #### getCostPerSecond

      ```
      public double getCostPerSecond()
      ```

      Gets the cost per second.

      Returns:
      :   the cost per second


    - #### setCostPerSecond

      ```
      protected void setCostPerSecond(double costPerSecond)
      ```

      Sets the cost per second.

      Parameters:
      :   `costPerSecond` - the new cost per second


    - #### getAllocationPolicy

      ```
      protected int getAllocationPolicy()
      ```

      Gets the allocation policy.

      Returns:
      :   the allocation policy


    - #### setAllocationPolicy

      ```
      protected void setAllocationPolicy(int allocationPolicy)
      ```

      Sets the allocation policy.

      Parameters:
      :   `allocationPolicy` - the new allocation policy


    - #### setVmm

      ```
      protected void setVmm(java.lang.String vmm)
      ```

      Sets the vmm.

      Parameters:
      :   `vmm` - the new vmm


- Overview
- Package
- Class
- Use
- Tree
- Deprecated
- Index
- Help

- Prev Class
- Next Class

- Frames
- No Frames

- All Classes

- Summary:
- Nested |
- Field |
- Constr |
- Method

- Detail:
- Field |
- Constr |
- Method
